# Magnetization Processes in Metallic Glass Based on Iron of FeSiB Type

**DOI:** 10.3390/ma15249015

**Published:** 2022-12-16

**Authors:** Zbigniew Stokłosa, Piotr Kwapuliński, Małgorzata Karolus

**Affiliations:** Institute of Materials Science, University of Silesia, 75-go Pułku Piechoty 1a, 41-500 Chorzów, Poland

**Keywords:** amorphous alloys, soft magnetic materials, magnetization processes, XRD analysis

## Abstract

In the present paper, the magnetization processes in amorphous alloys based on iron are discussed in detail. Our main goal was to measure the stabilization energy connected with the presence of microvoids (frozen during rapid cooling from the liquid phase) and to determine the interaction energy of relaxators with spontaneous magnetization vector (the so-called *w_N_* Neel) for amorphous Fe_78_Si_13_B_9_ alloys. A structural analysis of the alloys using X-ray measurements at the subsequent stages of crystallization was also performed.

## 1. Introduction

Iron-based amorphous alloys, due to their unique properties, are an interesting group of soft magnetic materials [1,2,3,4,5]. The magnetic parameters of these alloys are much better in comparison to silicon steels, and can be significantly improved by applying a controlled thermal treatment. [6,7,8]. Improvement of soft magnetic properties can be obtained by nanocrystallization or by formation of the so-called relaxed amorphous phase [9,10,11,12,13,14,15]. Magnetization processes of amorphous alloys based on iron are complex processes and depend on many elements.

The thermodynamic free energy of crystalline ferromagnetic samples, according to Morrish [16], is the sum of following components (with the accuracy of the additive constant *F*_0_):

(1) $$F=F_{0}+F_{H}+F_{D}+F_{K}+F_{\sigma }+F_{e}+F_{S}$$

where $F_{H}$ is the energy connected with the magnetization of the sample in an applied magnetic field *H*, $F_{D}$ is the energy connected with the magnetization of sample in their own magnetic field, $F_{K}$ is the magnetocrystalline anisotropy energy, $F_{\sigma }$ is the magnetoelastic energy, $F_{e}$ is the exchange energy, and $F_{S}$ is the stabilisation energy.

In amorphous soft magnetic materials, magnetoelastic energy and stabilisation energy, connected with the presence of relaxators, play a dominant role. The relaxators are atoms paired in the presence of free volumes (so-called microvoids) [17,18], frozen during rapid cooling from the liquid phase. In the case of nanocrystalline materials, the magnetocrystalline anisotropy energy dominates.

The main goal of the present work is to determine the stabilization energy connected with the presence of microvoids and the interaction energy of relaxators with spontaneous magnetization vectors (the so-called *w_N_* Neel) for amorphous Fe_78_Si_13_B_9_ alloys.

Magnetic delay, also called magnetic viscosity, originally observed in crystalline materials, is related to the migration of atoms of impurities or defects in the crystal lattice. This phenomenon also occurs in structurally disordered materials—in amorphous alloys. In the case of these materials, the delay phenomenon is caused by the directional ordering of atom pairs near the so-called free volumes [17,18]. Several phenomena caused by magnetic viscosity are known, including:

- Magnetic susceptibility disaccomodation;
- The phenomenon of magnetization delay with step changes of the constant magnetizing field (the so-called ∆H effect, caused by the directional ordering of the relaxators and fluctuation delays);
- The appearance of additional magnetic losses during their measurement with an alternating magnetic field (apart from the magnetic hysteresis and eddy currents loss);
- Pawlek effect (weak dependence of magnetic permeability versus magnetic field in the area of weak magnetizing fields);
- Perminwar effect (narrowing of the central part of the hysteresis loop caused by the Pawlek effect).

## 2. Experiment

In order to determine the condition of the samples in the as-quenched state and after 1 h annealing at different temperatures, X-ray diffraction (PANalytical Empyrean diffractometer) and high-resolution electron microscope (JEM 3010B) investigations were carried out. The isochronal resistivity curves (measured at room temperature) versus one hour annealing temperature were also determined to observe the decrease of resistivity connected with the crystallization process. The samples were annealed in a vacuum at a temperature range of 300–800 K with 25 K step.

The optimization temperature *T*_op_ (temperature of heat treatment that leads to obtaining the best soft magnetic properties) was determined from isochronal relative magnetic permeability curves versus 1 h annealing temperature (measurements were made at room temperature) as the temperature at which the permeability reaches its maximum.

Magnetic aftereffects after 1 h annealing were also measured. Quantity $\frac{\Delta \mu }{\mu }=\frac{\mu \left(t_{1}\right)-\mu \left(t_{2}\right)}{\mu \left(t_{1}\right)}\cdot 100\%$, where $t_{1}$ and $t_{2}$ denote times after demagnetization for 30 s and 1800 s, respectively. The above measurements were carried out by applying a precision Agilent RLC bridge.

In order to determine the stabilization energy connected with the presence of microvoids, the primary magnetization curves were measured. These curves were determined by applying a static magnetization field for different times after demagnetization. The curves were extrapolating for times t→0 and t→∞. Primary magnetization curves were obtained by applying the LikeShore fluxmeter. The stabilization energy was determined numerically by integration-obtained curves in a *H* = *f*(*B*) coordinate system. These investigations were carried out for samples in the as-quenched state.

The interaction energy between the relaxator and spontaneous magnetization vector(*w_N_*) can be determined approximately by estimating the concentration of relaxators (*c*). Concentration of relaxators was estimated from the vacancy malting model [19,20,21] and the multiplicity of increasing of the magnetic permeability after optimization annealing at *T*_op_ temperature and in the as-quenched state.

## 3. Results

**Figure 1:**
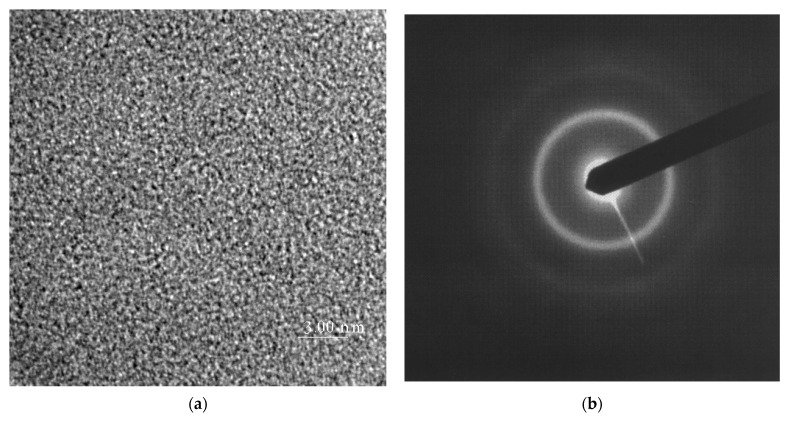
HREM image (**a**) and the corresponding electron diffraction pattern (**b**) for Fe_78_Si_13_B_9_ alloy after annealing at *T*_op_ temperature (623 K/1 h).

**Figure 2:**
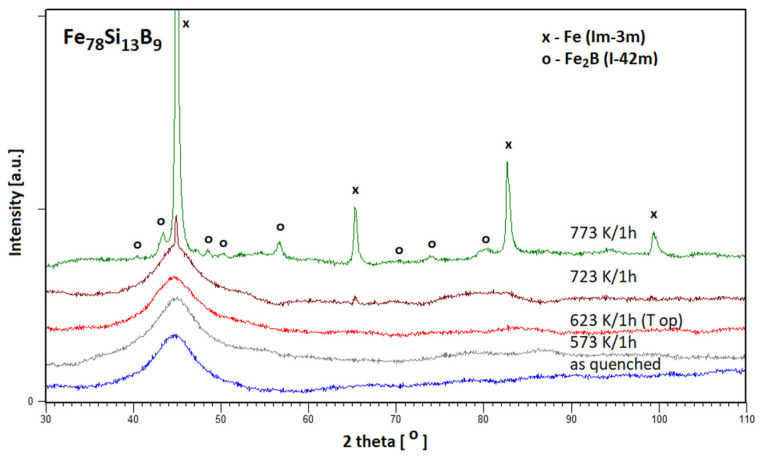
The X-ray diffraction patterns obtained for investigated alloys as-quenched and annealed at *T*_a_ = 573 K/1 h, *T*_a_ = *T*_op_ = 623 K/1 h, *T*_a_ = 723 K/1 h and at *T*_a_ = 773 K/1 h.

The structural investigations using X-ray diffraction and HREM are shown in Figure 1 and Figure 2. Figure 1 presents the HREM image and electron diffraction pattern after annealing at *T*_op_ temperature.

The X-ray diffraction patterns obtained for the investigated alloys as-quenched and annealed at *T_a_* = 573 K/1 h, *T*_a_ = *T*_op_ = 623 K/1 h, *T_a_* = 723 K/1 h (where the first step of crystallization is observed) and at *T*_a_ = 773 K/1 h are presented on Figure 2. The phase analysis performed with using the ICDD PDF4+ 2016 data base shows presence of Fe (Im-3m) and Fe_2_B (I-42m) phases.

**Figure 3:**
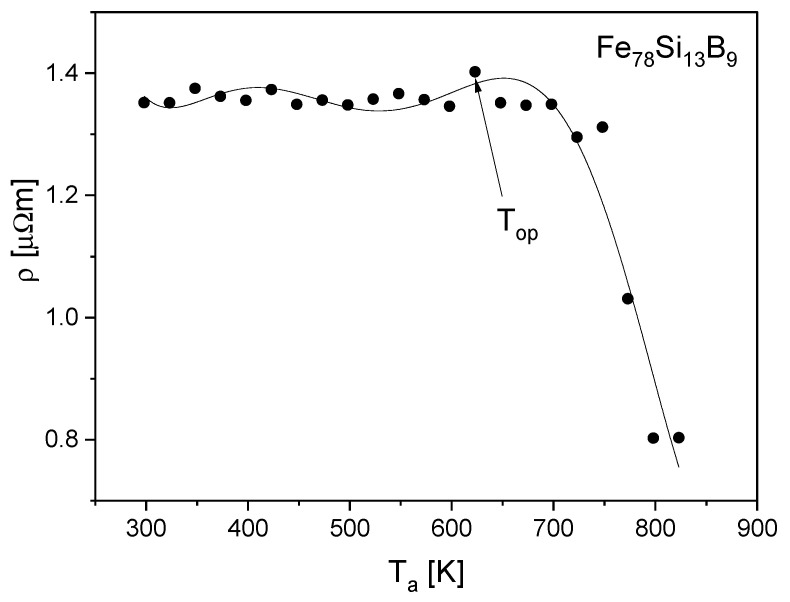
Isochronal electrical resistivity curves versus 1-h annealing temperature.

Careful examinations of the microstructure of the optimized samples (annealed at *T*_op_) carried out by applying X-ray diffraction and high-resolution electron microscopy do not show any traces of nanostructure, which means that optimization takes place in the relaxed amorphous phase. This fact is also confirmed by the isochronal electrical resistivity curves after 1-h annealing (measured at room temperature). A drastic decrease of resistivity connected with the crystallization process occurs above the *T*_op_ temperature (Figure 3).

The results of the structural analysis obtained by use of the Rietveld refinement [22] as a part of PANalytical High Score Plus 4.0 software [23] are presented in Table 1. During the annealing process, we observed the creation of the microcrystalline Fe phase and nanocrystalline Fe_2_B phase with the crystallite size in a range of 144 Å.

**Figure 4:**
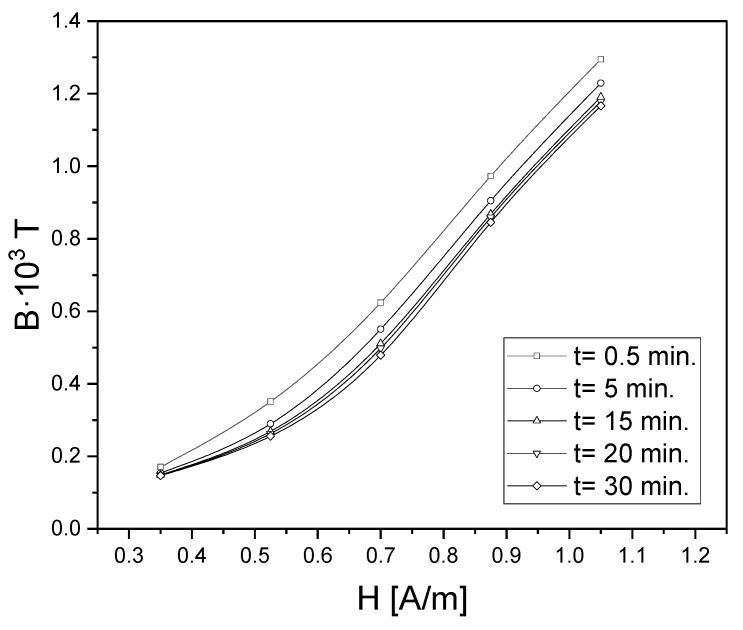
Isochronous curves *B*(*H*,*t*) for ti = const (counted from the moment of demagnetization) for the Fe_78_Si_13_B_9_ sample.

Based on the the magnetic susceptibility disaccomodation curves obtained for different magnetizing fields, the so-called isochronous *B*(*H*,*t*) curves are presented in Figure 4. Each of the *B*(*H*) curves corresponds to a certain time *t*, counted from the moment of demagnetization. The horizontal distance between the extreme curves: the curve *B*(*H*,0), obtained by extrapolation to *t* = 0, and the curve *B*(*H*,*t*), corresponding to the moment *t*, is called the viscosity field. This value tells us how much to increase the applied magnetic field after a certain time *t* in order to obtain the same magnetic induction *B* as for *t* = 0 (Figure 4). There were exemplary isochronous *B*(*H*,*t*) curves for time ti = const (time measured after the end of demagnetization) for the Fe_78_Si_13_B_9_ sample. The phenomenon of magnetic susceptibility disaccomodation was investigated using a constant (static method) or alternating magnetic field (dynamic method).

**Figure 5:**
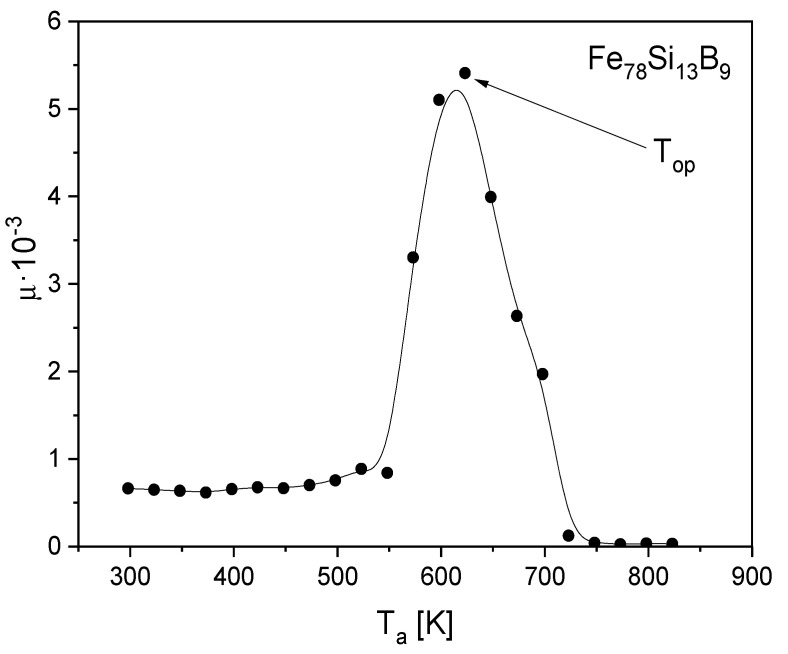
Isochronal magnetic permeability curves versus the 1 h annealing temperature.

Figure 5 presents the isochronal magnetic permeability curves versus the 1 h annealing temperature. The temperature at which the magnetic permeability reaches its maximum is defined as the optimization temperature (*T*_op_).

**Figure 6:**
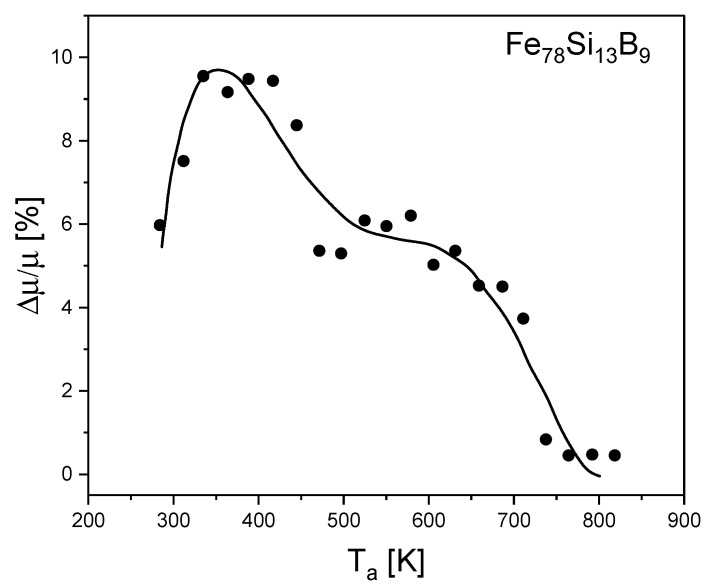
Magnetic aftereffects curves versus the 1 h annealing temperature.

The magnetic aftereffects curves versus the 1 h annealing temperature are presented in Figure 6. The quantity Δ*μ*/*μ* is proportional to the concentration of relaxators.

**Figure 7:**
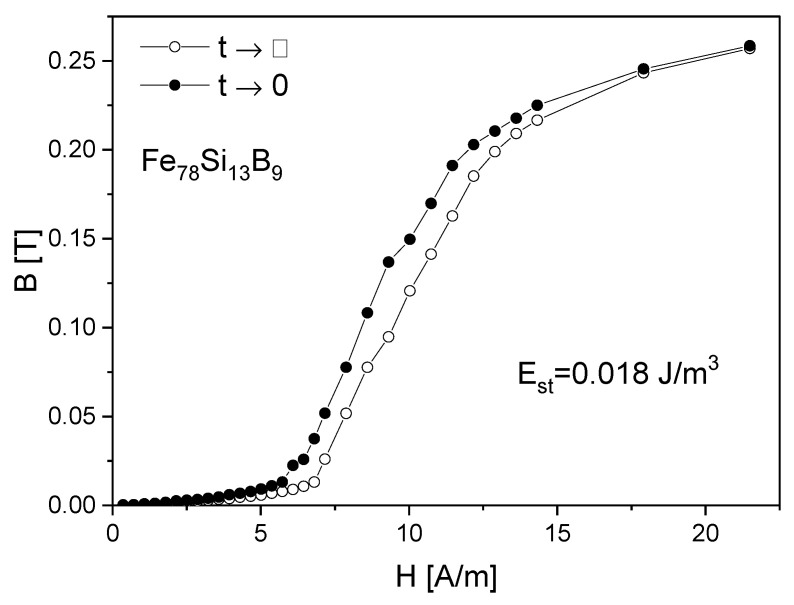
The *B*(*H*,*t*) curves obtained by static measurements, extrapolating to times t→0 and t→∞ after demagnetization.

Figure 7 presents the primary magnetization curves *B*(*H*,*t*) obtained by static measurements, extrapolating to times t→0 and t→∞. After integration in the *H* = *f*(*B*) coordinate system, the value $E_{st}=0.018$ J/m^3^ was obtained.

## 4. Discussion

As it was shown based on the X-ray and microscopic examinations, nanocrystallites are not observed after annealing at the *T*_op_ temperature. The magnetic permeability reaches a maximum at about 5000 and increases seven times in comparison to the as-quenched state. The resistivity, after annealing at *T*_op_, is close to the initial state, and it decreases after annealing above 723 K, which is connected to the appearance of the crystalline phase. Simultaneously, a significant decrease of magnetic permeability disacommodation (magnetic aftereffects) was observed. The magnetic aftereffects are proportional to the concentration of microvoids, i.e., the free volume frozen during the rapid cooling from the liquid phase. This is connected with the disappearance of microvoids and the formation of the so-called relaxed amorphous phase. Thus, the optimization of the soft magnetic properties takes place in the relaxed amorphous phase. After annealing at higher temperatures, the soft magnetic properties decrease due to the presence of crystalline phases and magnetic hardening of the material.

**Figure 8:**
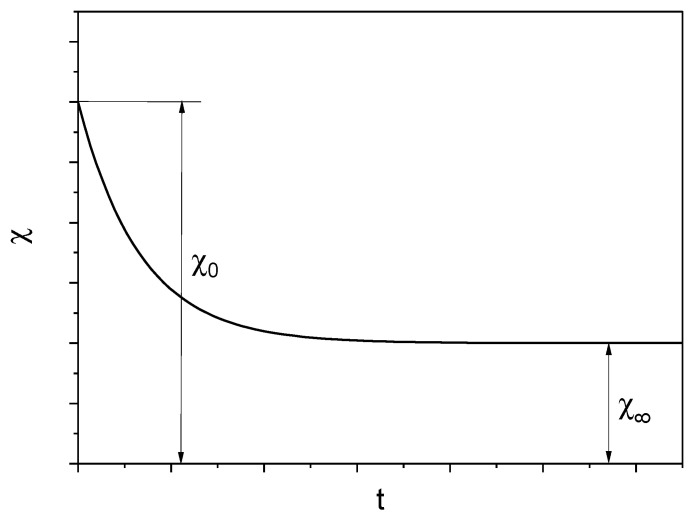
Disaccomodation curve of magnetic susceptibility χ from time *t*, χ_0_—magnetic susceptibility at time *t* = 0, χ∞—magnetic susceptibility for *t* → ∞ [24].

Magnetic susceptibility disaccomodation (magnetic aftereffects, MAE) is the most studied magnetic delay effect. It is caused in magnetic amorphous materials by reversible structural relaxations related to small displacements of atoms. Disaccomodation of magnetic susceptibility consists of decreasing the value of magnetic susceptibility of a sample demagnetized with an alternating current over time, with an amplitude descending to zero [18,24,25]. A typical magnetic susceptibility deaccomodation curve (magnetic susceptibility relaxation) is shown in Figure 8.

The reduction of magnetic susceptibility in metallic glasses according to H. Kronmüller is related to the ordering of atom pairs inside the domain walls. It consists of small shifts of groups of neighboring atoms. The stabilization of domain walls is caused by magnetic interactions. Moving of atom pairs tends to align their axes parallel to the direction of spontaneous magnetization.

**Figure 9:**
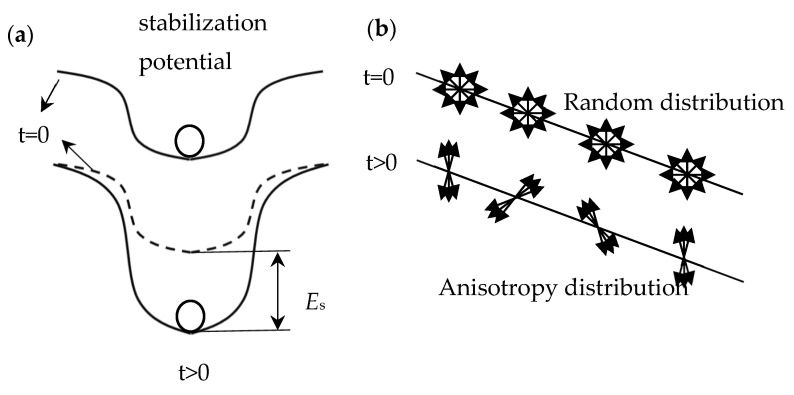
Diagram of the formation of the stabilization potential of the domain wall over time (**a**), diagram of the model of the magnetic delay phenomenon caused by the reorientation of the axis of pairs of atoms inside the domain wall (**b**) [24].

In time *t* = 0, the axes of atom pairs are positioned randomly, as shown in Figure 9a,b.

After time *t*, the distribution of the pairs of axes inside the domain wall is fan-shaped, the direction of which rotates according to the rotation of the spins inside the domain wall. When changing the direction of the axes of pairs of atoms, the total energy of the magnetic interactions is decreased, which leads to the formation of the so-called potential of domain wall stabilization, and in turn any change of this potential between time *t* = 0 and *t* causes a decrease of magnetic susceptibility [24].

**Figure 10:**
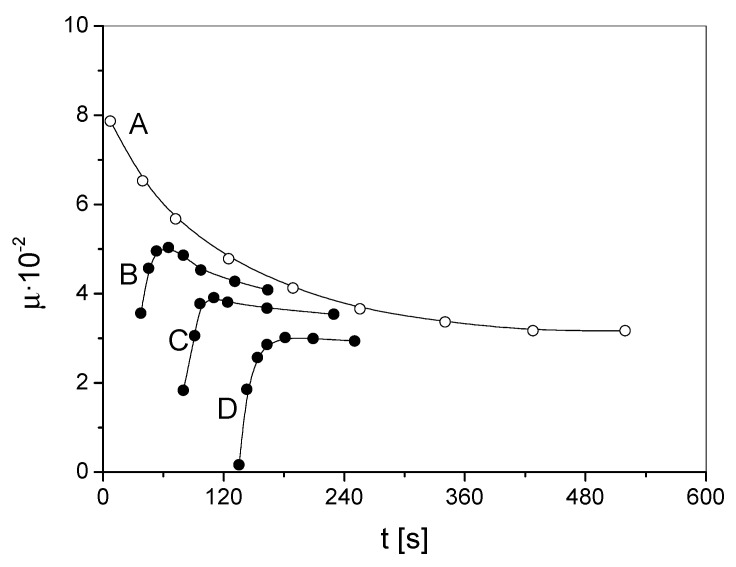
Magnetic permeability μr from time *t* after demagnetization; Webb–Ford effect in silicon steel, curve A for time *t* = 0 from the time of demagnetization, curves B, C, D—for later times ti.

In measurements in which the sample is magnetized with an alternating magnetic field, there is the so-called Webb–Ford effect for higher magnetic field strengths. In the case of very weak magnetic fields (for αFe-C of 1 mOe), the curve captured in the alternating field follows the same course as the curve obtained statically. For fields of about 10–100 mOe, when the sample is magnetized continuously, the permeability graph behaves slightly differently—its beginning will be consistent with the static curve, and then runs much higher than it (Figure 10, curve A). If a relatively short time t1 elapses after demagnetization, then the magnetic permeability, and thus the magnetic susceptibility, instead of decreasing monotonically with time, will first start to increase and only after passing a certain maximum will decrease monotonically (Figure 10, curves B, C).

The phenomenon of magnetic delay can be caused by processes such as [24]:

- Directional arrangement of point relaxators;
- The Zener mechanism, consisting of the directional ordering of pairs of substitution atoms;
- The so-called magnetic diffusion of point defects or foreign atoms;
- Thermally activated dislocation movement;
- Magnetic migratory relaxation related to grain boundaries.

In a stabilized sample, the interstitial atom distribution is the equilibrium distribution with respect to the existing domain structure. After demagnetization, a new domain structure appears in which the distribution of interstitial atoms (impurities) is no longer an equilibrium distribution. As a result, the permeability after demagnetization is much higher than in the case of a stabilized sample (after time *t* → ∞ from demagnetization). The directional ordering of the interstitial atoms leads to a new distribution of atoms, corresponding to the new domain structure. The bottom of the potential, in which the domain wall is located, is “deepened” (Figure 9a), which in turn reduces the magnetic susceptibility, i.e., its disaccomodation [25].

It is worth noting that in the time–temperature instabilities of amorphous materials, relaxation effects are observed even at relatively low temperatures. Structural relaxation in amorphous materials strongly influences such magnetic properties as additional magnetic losses, magnetic susceptibility, and coercive field strength. Analysis and interpretation of relaxation phenomena in amorphous alloys is more complex than in the case of bodies with a crystalline structure, where these phenomena result from the presence of precisely defined structural defects, i.e., vacancies or interstitial atoms. The relaxation processes in crystalline materials correspond to discrete energy values and a narrow temperature range.

Magnetic permeability, as one of many quantities sensitive to structure and phase transitions, in the first approximation depends on magnetoelastic energy, stabilization energy, magnetocrystalline energy and saturation magnetization [26,27,28,29,30,31].

### 4.1. Magnetoelastic Energy

Magnetoelastic energy, among others, depends on the magnetostriction coefficient and internal stresses. In the quenched amorphous alloys, the longitude magnetostriction coefficient in the saturation magnetization field is significantly higher than the magnetostriction coefficient after annealing at *T*_op_. For alloys with compositions determined by obtaining *T*_op_ in the nanocrystalline phase, it is higher in the relaxed amorphous phase. Initially it was shown that the effective magnetostriction coefficient of composite materials consisting of the amorphous and nanocrystalline phases in an amount *α* is additive quantity and amounts to [32]:

(2) $$\lambda_{s}=\left(1-\alpha \right)\lambda_{s}^{am}+\alpha \lambda_{s}^{c}$$

where $\lambda_{s}^{am}$, $\lambda_{s}^{c}$ are the magnetostriction coefficients of the amorphous and crystalline phase, respectively. Later, it was shown that the effective magnetostriction coefficient also depends on specific surface area *S*/*V* of the nanocrystalline phase in an amorphous matrix [33]:

(3) $$\lambda_{s}=\alpha \lambda_{s}^{c}+\left(1-\alpha \right)\left(\lambda_{s}^{am}+k\alpha \right)+\alpha \lambda_{s}^{s}S/V$$

where $\lambda_{s}^{s}$ is the magnetostriction coefficient characterising the surface between amorphous and nanocrystalline phases, *k* is the parameter describing changes of magnetostriction coefficient of the amorphous phase during crystallization.

### 4.2. Stabilization Energy

In the case of the presence in magnetic materials of diffused elements interacting with the vector of spontaneous magnetization, additional stabilization energy occurs. In magnetic materials obtained by the melt spinning method, the diffusion elements are (according to Kronmüller [18]) atom pairs with presence of microvoids. Then, stabilization energy after time *t*, on the assumption of one relaxation time, is proportional to the expression:

(4) $$E_{st}\left(t\right)\approx \frac{w_{N}^{2}C}{3k_{B}T}\left(1-e^{-\frac{t}{\tau }}\right)$$

where *w_N_* is the interaction energy of spontaneous magnetization vector with the magnetic relaxators, *c* is the concentration of the relaxators, *k_B_* is the Boltzmann constant and *T* is the absolute temperature.

### 4.3. Magnetocrystalline Energy

Magnetocrystalline energy depends on magnetocrystalline anisotropy constant *K*_1_ and induced anisotropy constant *K_u_*. This energy has a high influence on the magnetic properties of alloys, of which *T*_op_ is higher than the crystallization temperature *T*_x1_.

For the small-sized crystalline phase distributed in an amorphous matrix, the length of exchange interaction includes several nanocrystallites. According to Herzer [34,35], the length of exchange interaction can be expressed by:

(5) $$L_{0}=\varphi \sqrt{\frac{A}{K_{1}}}$$

where *A* is the exchange interaction constant, *φ* is the proportionality coefficient closed to one. If $K_{1}>K_{u}$, then the effective coefficient of magnetocrystalline anisotropy j $\underline{K}$ of the composite is equal:

(6) $$\underline{K}=\alpha K_{1}{\left(\frac{d}{L_{0}}\right)}^{6}$$

where *d* is the arithmetic average diameter of nanocrystallites, *α* is the volume fraction of nanocrystalline phase. When $K_{1}\ll K_{u}$ then $\underline{K}$ is equal to:

(7) $$\underline{K}=K_{u}+\frac{1}{2}\alpha \sqrt{K_{u}+K_{1}}{\left(\frac{d}{L_{0}}\right)}^{3}$$

The above-mentioned mechanisms have an influence on the value of relative magnetic permeability, which can be estimated from the formula [19]:

(8) $$\mu_{r}=\frac{3k_{B}TA^{3}J_{s}^{2}}{2\mu_{0}\left\{3k_{B}Td^{6}K_{1}^{4}+\frac{9}{2}\lambda_{s}\sigma A^{3}k_{B}T+w_{N}^{2}cA^{3}\left[1-e^{-\frac{t}{\tau }}\right]\right\}}$$

where the temperature *T*_op_ is below the crystallization temperature, and is taking into account the change in two terms in the denominator and the change of *J_s_*, hence $\mu_{r}^{opt}$ after annealing at *T*_op_ can be several times higher than $\mu_{r}^{asq}$ in the as-quenched state.

It must be noted that individual parts of the energy which have an influence on $\mu_{r}$ are mutually dependent. For example, when $\lambda_{s}$ strongly depends on concentration of microvoids, mechanical stresses are also dependent on this value, and *J_s_* is the function of microvoid concentration and nanocrystalline phase quantity.

As mentioned above, the dominant role in the magnetization processes of investigated amorphous materials is shown in the stabilization energy connected with the presence of relaxators. [18]. For the Fe_78_Si_13_B_9_ alloy, it received the value 0.018 J/m^3^. This value is numerically equal to the constant preceding the function $\left(1-e^{-\frac{t}{\tau }}\right)$ in the Equation (4):

(9) $$E_{0}=\frac{w_{N}^{2}c}{3k_{B}T}$$

By estimating the concentration of the relaxators (*c*), the interaction energy between the relaxator and spontaneous magnetization vector (*w_N_*) can be determined. Using the vacancy melting model [20,21,26] and increasing the magnetic permeability after annealing at *T*_op_ in comparison to permeability in the as-quenched state, the concentration of relaxators was estimated. In the present paper, the value 8.4 × 10^−23^ J/relaxator for *w_N_* was obtained. In 1956, values of 6.4 × 10^−23^ J/relaxator for C atoms in αFe and 5.7 × 10^−23^ J/relaxator for N atoms in αFe were obtained by Nèel. In 1997, for the pair atoms C-C in αFe, the value 5.5 × 10^−23^ J/relaxator was obtained [36]. The value *w_N_*, for the investigated alloy, is about 12-times higher than for the interstitial solution C and N in iron. It should be concluded that the free volumes have a large role in the processes of magnetization of the amorphous materials.

It should be noted that at present, in traditional materials such as silicon steels, effects connected with the presence of microvoids are negligibly small in comparison with the magnetocrystalline energy due to the applied technological processes.

## 5. Conclusions

1. The optimization of the soft magnetic properties is obtained by the formation of the relaxed amorphous phase (Figure 1, Figure 2 and Figure 3).
2. After the annealing process, microcrystalline Fe and nanocrystalline Fe_2_B phase with the crystallite size in a range of 144 Å were created.
3. The improvement of soft magnetic properties in the relaxed amorphous phase is connected to the disappearance of the free volume (Figure 3 and Figure 5).
4. Magnetization processes in iron-based amorphous FeSiB alloys mainly depend on stabilization energy of the domain wall motion. In the present paper, the value $E_{st}=0.018$ J/m^3^ was obtained.

5. The obtained interaction energy between the spontaneous magnetization vector and relaxators is, in the case of amorphous materials, an order of magnitude higher than for solid solutions in crystalline materials.

## Figures and Tables

**Table 1:** Structural characterization of Fe_78_Si_13_B_9_ alloy annealed at *T*_a_ = 773 K/1 h after crystallization.

Fe				Fe_2_B			
Theoretical (ICDD PDF4 + Card: 01-087-0721)	Refined (RR) a [Å]	Crystallite Size D [Å]	Lattice Strain η [%]	Theoretical (ICDD PDF4 + Card: 96-101-0475)	Refined (RR) a/c [Å]	Crystallite Size D [Å]	Lattice Strain η [%]
a = 2.8662 Å Space Group: Im-3m Crystallographic System: Cubic	a = 2.8529(2)	>1000	0.12	a = 5.0990 Å c = 4.2400 Å Space Group: I-42m Crystallographic System: Tetragonal	5.1196(3) 4.2131(9)	144	0.15

## Data Availability

All data generated or analyzed during this study are included in this published article.
